# Time trends of neonatal mortality by causes of death in Shenyang, 1997–2014

**DOI:** 10.18632/oncotarget.7670

**Published:** 2016-02-24

**Authors:** Qi-Jun Wu, Li-Li Li, Jing Li, Chen Zhou, Yan-Hong Huang

**Affiliations:** ^1^ Department of Clinical Epidemiology, Shengjing Hospital of China Medical University, Shenyang, China; ^2^ Department of children's health prevention, Shenyang Women and Children Health Care Centre, Shenyang, China; ^3^ Department of science and education, Shenyang Women and Children Health Care Centre, Shenyang, China; ^4^ Department of information statistics, Shenyang Women and Children Health Care Centre, Shenyang, China

**Keywords:** annual percent change, neonatal mortality rate, Shenyang, trend

## Abstract

To investigate the rate and time trends of neonatal mortality from 1997 to 2014 in Shenyang, which were previously rarely reported upon by developing countries, data on 4719 neonatal deaths (0–28 days) and 970,583 live births from the Shenyang Women and Children Health Care Centre were analyzed. Neonatal mortality rates (per 1000 live births), percent change, and annual percent change (APC) were calculated. During the observation period, neonatal mortality in Shenyang significantly decreased by 7.04%, 8.33%, and 5.35% per year overall, in urban and rural areas, respectively. When grouped by category of neonatal death, the time trends of three categories showed statistically significant decreases: congenital malformations (APC = −9.97%), diseases of the perinatal period (APC = −6.04%), and diseases of the respiratory system (APC = −8.52%). Congenital malformations, diseases of the respiratory system, and diseases of the nervous system and sense organs were the three major contributors to the aforementioned decreasing trend, which accounted for 58.71% in overall areas. Among selective causes of neonatal death, the neonatal mortality rates of pneumonia, congenital heart disease, preterm birth and low birth weight, birth asphyxia, and intracranial hemorrhage of the newborn significantly decreased 7.87%, 7.32%, 2.47%, 11.04%, and 10.68% per year, respectively. In summary, neonatal mortality rates decreased in Shenyang during the 17-year study period. Future studies are warranted to further investigate the factors contributing to the neonatal mortality trends in China.

## INTRODUCTION

Recent data from the United Nations Children's Fund (UNICEF), entitled, A Promise Renewed: 2015 Progress Report demonstrated that since 2000, when governments committed to achieving the Millennium Development Goals (MDGs), the lives of 48 million children under the age of five have been saved [1]. Additionally, in contrast to more than 12 million children who died in 1990, 6.6 million children died before reaching their fifth birthday in 2012, which was a sharp decrease. However, these results are still insufficient when compared to one of the most prominent goals for 2015 (MDG-4), which aims to reduce the child mortality rate by two-thirds from the level reported in 1990 [2].

The neonatal mortality rate is an important indicator used for determining the effectiveness of public health issues, including maternal and child health care services as well as for comparing countries with respect to welfare initiatives [3]. It allows researchers to monitor time and territorial trends in mortality, and consequently, to plan and introduce organizational changes to the health care system [4]. The neonatal mortality rates varied in most countries. In general, neonatal mortality rates are high in Africa and South-Central and Western Asia; intermediate in Eastern Asia, South America, the Caribbean, and Australia/New Zealand; and low in North America and Europe [5]. Besides effective medical technology, better access to pre- and postnatal care for all socioeconomic groups and better nutrition [6], the factors that contribute to the international variation in neonatal mortality rates largely stem from differences in the development levels of these countries. Similarly, in China, a wide gap exists in terms of people's income and health status between urban and rural areas and the gap explains the regional differences in children's health status and survival between urban and rural areas [7]. During the last year there were attempts to reach the 2015 MDG deadline on reducing child mortality as well as to provide valuable evidence from developing countries; therefore, we carry out this study to analyze the rate and time trends of neonatal mortality in Shenyang on the basis of a relatively long observation period (1997–2014), with special attention paid to stratifying these results by areas.

## RESULTS

**Figure 1:**
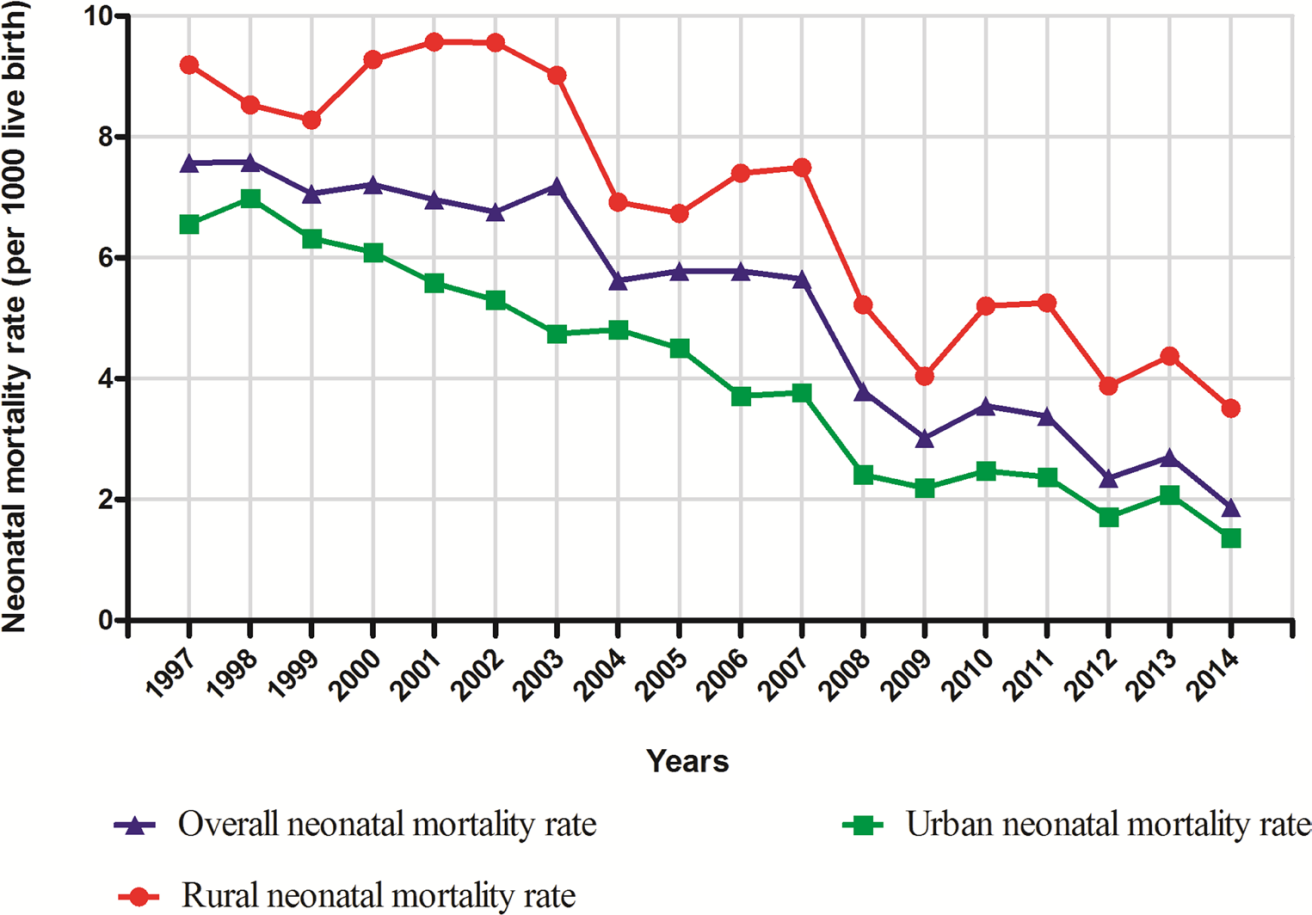
Trends in neonatal mortality rate (per 1000 live births) by areas in Shenyang, 1997–2014

During the 17-year interval, there were 970,583 live births and 4719 neonatal deaths in Shenyang (Table 1). When stratified by areas, the neonatal mortality rates of urban areas were significantly lower than that of rural areas (3.67 *versus* 6.68 per 1000 live births). Figure 1 visually depicts the time trend and the overall neonatal mortality rate in Shenyang during the time period 1997–2014. The overall neonatal mortality rate was 4.86 per 1000 live births (Table 2). The overall mortality of neonate significantly decreased by 75.34% from 7.57 to 1.87 per 1000 live births, or 7.04% per year (95% CI = −8.60%–−5.45%). When stratified by urban and rural areas, the neonatal mortality rate has decreased systematically by 8.52% (95% CI = −9.67%–−7.34%) in urban areas, and slightly more slowly in rural areas with APC = −5.26% (95% CI = −6.85%–−3.64%) (Table 3).

**Table 1:** Neonatal mortality rates in urban and rural areas of Shenyang during 1997–2014

Year	Urban and rural areas			Urban areas^†^			Rural areas^†^		
	Live births	No.	Rate^*^	Live births	No.	Rate^*^	Live births	No.	Rate^*^
1997	48628	368	7.57	30022	197	8.49	18606	171	9.19
1998	45881	348	7.58	27943	195	8.41	17938	153	8.53
1999	42474	300	7.06	26416	167	7.87	16058	133	8.28
2000	44245	319	7.21	28723	175	8.18	15522	144	9.28
2001	43244	301	6.96	28306	158	7.88	14938	143	9.57
2002	46163	312	6.76	30367	161	7.05	15796	151	9.56
2003	36015	259	7.19	15400	73	6.43	20615	186	9.02
2004	46240	260	5.62	28473	137	7.02	17767	123	6.92
2005	52972	306	5.78	22674	102	6.35	30298	204	6.73
2006	52237	302	5.78	22896	85	5.20	29341	217	7.40
2007	61111	345	5.65	30251	114	5.42	30860	231	7.49
2008	59163	224	3.79	30255	73	4.30	28908	151	5.22
2009	59232	179	3.02	32488	71	3.26	26744	108	4.04
2010	57523	204	3.55	34823	86	3.36	22700	118	5.20
2011	58303	197	3.38	37919	90	3.14	20384	107	5.25
2012	69469	163	2.35	49121	84	2.69	20348	79	3.88
2013	67856	183	2.70	49533	103	2.93	18323	80	4.37
2014	79827	149	1.87	61002	83	2.39	18825	66	3.51
Total	970583	4719	4.86	586612	2154	3.67	383971	2565	6.68

* Neonatal mortality rates were expressed as per 1000 live births.

† Urban areas included five districts (He Pin, Shen He, Da Dong, Huang Gu, and Tie Xi); Rural areas included eight districts (Liao Zhong, Kang Pin, Fa Ku, Xin Ming, Shen Bei, Hun Nan, Yu Hong, and Su Jiatun).

**Table 2:** Trends in neonatal mortality rate in Shenyang in 1997–2014

Cause of death	1997		2014		PC^†^ (%)	APC^†^ (%)	95% CI
	Case	Rate^*^	Case	Rate^*^			
Overall	368	7.57	149	1.87	−75.34	−7.04	−8.60, −5.45
Infectious and parasitic diseases	1	0.02	6	0.08	265.50	−2.37	−7.41, 2.94
Congenital malformations	121	2.49	21	0.26	−89.43	−9.97	−12.04, −7.84
Diseases of the perinatal period	201	4.13	95	1.19	−71.21	−6.04	−7.20, −4.81
Diseases of the nervous system and sense organs	1	0.02	2	0.03	21.83	−6.67	−14.44, 1.81
Diseases of the respiratory system	31	0.64	11	0.14	−78.38	−8.52	−12.31, −4.55
Disease of the digestive system	0	0	5	0.06	N/A	1.11	−7.70, 10.76
Accident	5	0.10	2	0.03	−75.63	−3.63	−11.84, 5.34
Others^§^	8	0.16	8	0.10	−39.08	−6.01	−12.92, 1.44

APC, annual percent change; CI, confidence interval; N/A, not available; PC, percent change.

* Neonatal mortality rates were expressed as per 1000 live births.

† Percent change and annual percent change between 1997 and 2014 was calculated by the neonatal mortality rate.

§ Others including endocrine, nutritional and metabolic diseases, blood and hematopoietic organ diseases, circulatory system diseases, urinary system diseases, leukemia and other neoplasms, and unknown or missing causes of deaths.

**Table 3:** Trends in neonatal mortality rate in Shenyang by urban and rural areas in 1997–2014

Cause of death	Urban area^‡^						95% CI	Rural area^‡^						95% CI
	1997		2014		PC^†^ (%)	APC^†^ (%)		1997		2014		PC^†^ (%)	APC^†^ (%)	
	Case	Rate^*^	Case	Rate^*^				Case	Rate^*^	Case	Rate^*^			
Overall	197	4.05	83	1.04	−74.33	−8.52	−9.67, −7.34	171	3.52	66	0.83	−76.49	−5.26	−6.85, −3.64
Infectious and parasitic diseases	1	0.02	4	0.05	143.67	−5.07	−12.41, 2.90	0	0	2	0.03	N/A	1.61	−4.24, 7.83
Congenital malfor mations	79	1.62	9	0.11	−93.06	−12.89	−14.90, −10.83	146	3	52	0.65	−78.30	−5.54	−6.93, −4.13
Disease of the perinatal period	97	1.99	55	0.69	−65.46	−6.67	−8.04, −5.27	104	2.14	40	0.5	−76.57	−5.16	−6.36, −3.95
Diseases of the nervous system and sense organs	0	0	0	0	N/A	−9.88	−18.08, −0.86	1	0.02	1	0.01	−39.08	−1.29	−8.16, 6.09
Diseases of the respiratory system	15	0.31	6	0.08	−75.63	−8.88	−13.95, −3.51	16	0.33	5	0.06	−80.96	−7.60	−11.81, −3.18
Disease of the digestive system	0	0	3	0.04	N/A	−2.47	−15.02, 11.94	0	0	2	0.03	N/A	−2.27	−9.84, 5.92
Accident	2	0.04	2	0.03	−39.08	−4.78	−13.63, 4.97	3	0.06	0	0	−100.00	0.4	−6.98, 8.36
Others^§^	3	0.06	4	0.05	−18.78	−5.54	−12.48, 1.95	5	0.1	4	0.05	−51.27	−4.30	−11.71, 3.72

APC, annual percent change; CI, confidence interval; N/A, not available; PC, percent change.

* Neonatal mortality rates were expressed as per 1000 live births.

† Percent change and annual percent change between 1997 and 2014 was calculated by the neonatal mortality rate.

‡ Urban areas included five districts (He Pin, Shen He, Da Dong, Huang Gu, and Tie Xi); Rural areas included eight districts (Liao Zhong, Kang Pin, Fa Ku, Xin Ming, Shen Bei, Hun Nan, Yu Hong, and Su Jiatun).

§ Others including endocrine, nutritional and metabolic diseases, blood and hematopoietic organ diseases, circulatory system diseases, urinary system diseases, leukemia and other neoplasms, and unknown or missing causes of deaths.

### Causal categories of neonatal death

**Figure 2:**
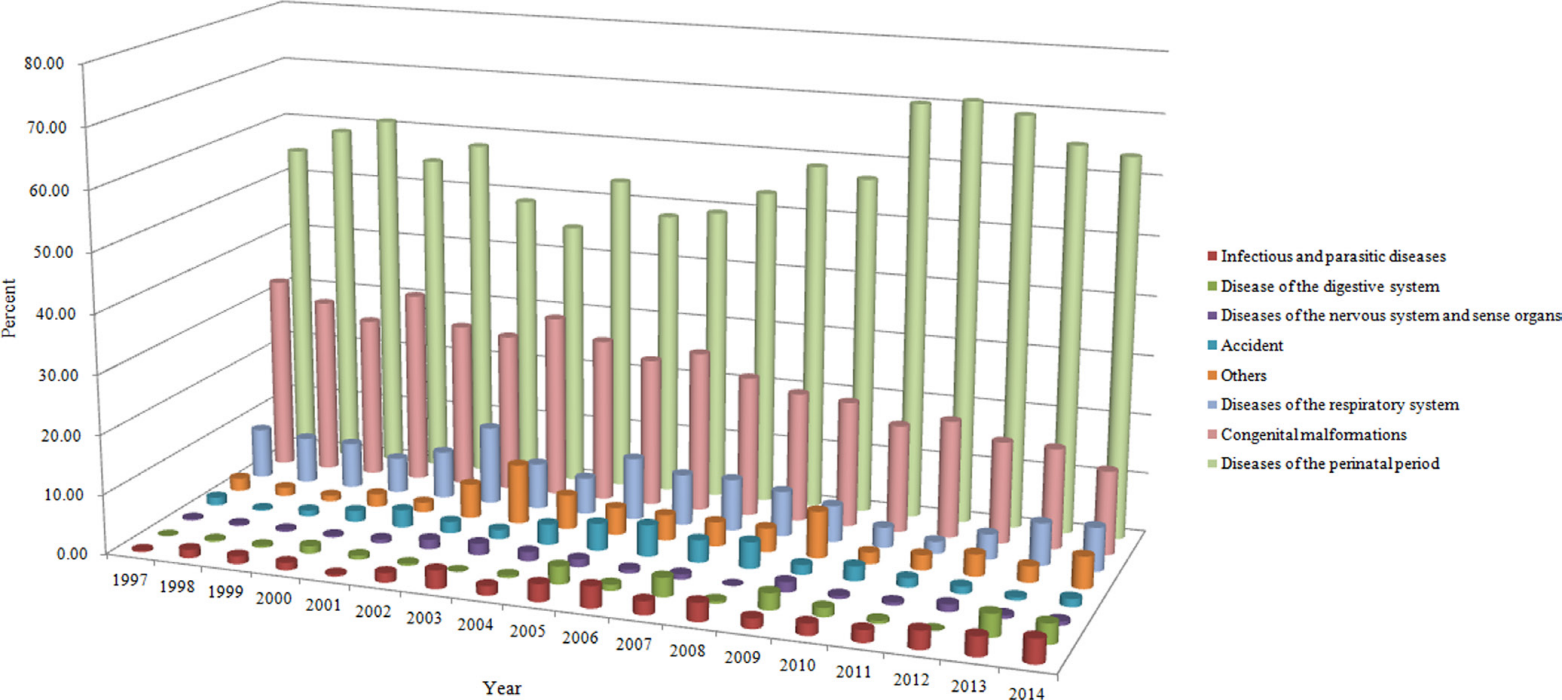
The proportions of categories of causes of neonatal mortality in urban and rural areas, 1997–2014

Casual categories of neonatal death and time trends were shown in Table 2. Neonatal mortality rates decreased significantly in 1997–2014 in the following categories: congenital malformations (APC = −9.97%, 95% CI = −12.04%–−7.84%), diseases of the perinatal period (APC = −6.04%, 95% CI = −7.20%–−4.81%), and diseases of the respiratory system (APC = −8.52%, 95% CI = −12.31%–−4.55%). The time trends of other casual categories of neonatal death showed non-statistical significance. Similar decreasing patterns were also observed in Table 3, which were stratified by urban and rural areas. The contribution rates of each category of death causes were provided in Table 4. Congenital malformations, diseases of the respiratory system, and diseases of the nervous system and sense organs were the three major contributors to the aforementioned decreasing trend, which accounted for 58.71% in urban and rural areas. Notably, diseases of the nervous system and sense organs were the second major contributors to the decreasing trend of neonatal mortality among urban areas, being responsible for 17.71% of the decrease. In contrast, this cause of neonatal death was the smallest contributor to the decreasing trend of neonatal mortality among rural areas, being responsible for 4.66% of the decrease. Figure 2 demonstrated the proportional causal categories of neonatal death.

**Table 4:** The relative contributions of each death causes by urban and rural areas in Shenyang, 1997–2014

Cause of death	Urban and Rural areas				Urban areas		Rural areas			
	Decreasing trend		Increasing trend		Decreasing trend		Decreasing trend		Increasing trend	
	β	Contribution rate (%)	β	Contribution rate (%)	β	Contribution rate (%)	β	Contribution rate (%)	β	Contribution rate (%)
Infectious and parasitic diseases	−0.024	5.36	–	–	−0.052	8.86	–	–	0.016	80
Congenital malformations	−0.105	23.44	–	–	0.138	23.51	−0.067	24.01	–	–
Diseases of the perinatal period	−0.062	13.84			−0.069	11.75	−0.053	18.99		
Diseases of the nervous system and sense organs	−0.069	15.4	–	–	−0.104	17.71	−0.013	4.66	–	–
Diseases of the respiratory system	−0.089	19.87	–	–	−0.093	15.84	−0.079	28.32	–	–
Disease of the digestive system	–	–	0.011	100	−0.025	4.26	−0.023	8.24	–	–
Accident	−0.037	8.26	–	–	−0.049	8.35	–	–	0.004	20
Others ^§^	−0.062	13.84	–	–	−0.057	9.71	−0.044	15.77	–	–

§ Others including endocrine, nutritional and metabolic diseases, blood and hematopoietic organ diseases, circulatory system diseases, urinary system diseases, leukemia and other neoplasms, and unknown or missing causes of deaths.

### Selective causes of neonatal death

Selective causes of neonatal death and time trends were demonstrated in Table 5. Except for septicemia and accidental asphyxia, the time trends of other selective causes of neonatal death showed statistically significant decreases. For example, between 1997 and 2014, the neonatal mortality rates of birth asphyxia, intracranial hemorrhage of the newborn, and pneumonia significantly decreased by −11.04%, 10.68%, and −7.87% per year, respectively. When stratified by urban and rural areas, similar patterns were still observed (Table 6).

**Table 5:** Trends in neonatal mortality rate of selective causes in Shenyang in 1997–2014

Selective causes of death	1997		2014		PC^†^ (%)	APC^†^ (%)	95% CI
	Case	Rate^*^	Case	Rate^*^			
Septicemia	1	0.02	6	0.08	265.50	−2.08	−7.53, 3.69
Pneumonia	29	0.60	8	0.10	−83.20	−7.87	−11.51, −4.09
Congenital heart disease	38	0.78	11	0.14	−82.37	−7.32	−9.65, −4.93
Preterm birth and low birth weight	77	1.58	66	0.83	−47.79	−2.47	−4.31, −0.59
Birth asphyxia	89	1.83	20	0.25	−86.31	−11.04	−11.98, −10.09
Intracranial hemorrhage of the newborn	28	0.58	3	0.04	−93.47	−10.68	−13.30, −7.99
Accidental asphyxia	5	0.10	2	0.03	−75.63	−3.63	−11.09, 4.45

APC, annual percent change; CI, confidence interval; PC, percent change.

* Neonatal mortality rates were expressed as per 1000 live births.

† Percent change and annual percent change between 1997 and 2014 was calculated by the neonatal mortality rate.

**Table 6:** Trends in neonatal mortality rate of selective causes in Shenyang by urban and rural area in 1997–2014

Selective cause of death	Urban area ^‡^						95% CI	Rural area ^‡^						95% CI
	1997		2014		PC^†^ (%)	APC^†^ (%)		1997		2014		PC^†^ (%)	APC^†^ (%)	
	Case	Rate^*^	Case	Rate^*^				Case	Rate^*^	Case	Rate^*^			
Septicemia	1	0.03	4	0.07	96.86	−5.07	−12.97, 3.55	0	0	2	0.11	N/A	1.61	−4.24, 7.83
Pneumonia	13	0.43	6	0.1	−77.29	−7.41	−12.75, −1.75	16	0.86	2	0.11	−87.65	−7.78	−11.42, −3.99
Congenital heart disease	21	0.7	4	0.07	−90.63	−10.86	−13.47, −8.18	17	0.91	7	0.37	−59.30	−3.34	−6.17, −0.43
Preterm birth and low birth weight	31	1.03	41	0.67	−34.91	−2.66	−5.11, −0.16	46	2.47	25	1.33	−46.28	−1.88	−3.94, 0.22
Birth asphyxia	47	1.57	11	0.18	−88.48	−11.31	−13.17, −9.41	42	2.26	9	0.48	−78.82	−10.68	−12.56, −8.77
Intracranial hemorrhage of the newborn	14	0.47	1	0.02	−96.48	−10.68	−14.94, −6.22	14	0.75	2	0.11	−85.88	−10.95	−14.47, −7.29
Accidental asphyxia	2	0.07	2	0.03	−50.76	−4.02	−13.49, 6.49	3	0.16	0	0	−100.00	0.3	−5.68, 6.66

PC, percent change; APC, annual percent change; CI, confidence interval.

* Neonatal mortality rates were expressed as per 1000 live births.

† Percent change and annual percent change between 1997 and 2014 was calculated by the neonatal mortality rate.

‡ Urban areas included five districts (He Pin, Shen He, Da Dong, Huang Gu, and Tie Xi); Rural areas included eight districts (Liao Zhong, Kang Pin, Fa Ku, Xin Ming, Shen Bei, Hun Nan, Yu Hong, and Su Jiatun).

## DISCUSSION

During the 17 years observational period, we observed a considerable decrease in neonatal mortality from 7.57 to 1.87 per 1000 live births, or 7.04% per year. Notably, the decreasing trend was slightly stronger in urban areas (8.52% per year) than rural areas (5.26% per year). Congenital malformations, diseases of the respiratory system, and diseases of the nervous system were the three major contributors to the aforementioned decreasing trend, which accounted for 58.71% in urban and rural areas. Among selective causes of neonatal death, significant decreasing trends were also observed in pneumonia, congenital heart disease, birth asphyxia, and intracranial hemorrhage of the newborn, as well as stratified by urban and rural areas.

The first and second leading cause of neonatal mortality in Shenyang at the present time is diseases of the perinatal period and congenital malformations. Deaths due to diseases of the perinatal period and congenital malformations have decreased from about 4.13 to 1.19 per 1000 live births and 2.49 to 0.26 per 1000 live births in the period between 1997 and 2014 in urban and rural areas, respectively (Table 1), which might be attributed to the big advances in obstetrics and neonatology during this period. The introduction and rapid dissemination of sonographic investigations during pregnancy and the subsequent changes in obstetric management of high-risk births are probably the main reasons for this improvement. In addition, pregnancies with prenatal problems were detected earlier and more often and thus the mothers could be transferred to specialized centres before giving birth [6, 8]. Moreover, the development of treatments for manageable disorders, such as congenital malformations of the heart, was improved upon considerably, which increases the survival rates of patients with these disorders [9, 10]. Additionally, since the difference between the development of urban and rural areas, the APC of congenital malformations and diseases of the perinatal period of urban areas was smaller than that of rural areas.

Among selective causes of neonatal mortality, except for preterm birth and low birth weight which belong to the diseases of perinatal period, birth asphyxia was the leading cause of neonatal mortality in urban and rural areas (Table 4). Previous studies suggested that decreasing neonatal mortality of birth asphyxia mainly resulted from high rates of hospital delivery [7]. Compared to hospital delivery, home delivery, mainly assisted by a village doctor or midwife without sufficient knowledge and skill, is strongly linked with poverty, poor transport connection or superstition [7]. In contrast to the 1.5% of neonatal deaths that were attributed to home delivery in urban areas, almost 25% of neonatal deaths resulted from home delivery in Chinese rural areas between 2003 and 2006. Therefore, during the past decades, the Chinese government has been implementing a subsidy project to allow rural pregnant women to give birth in hospitals; thereby, reducing the neonatal mortality through improving rural hospital delivery rates [11]. Furthermore, the considerable decrease in the neonatal mortality of pneumonia during the observation period could be attributed to substantial improvements in the medical treatment administered in neonatal intensive care units, and to improved care during the perinatal period [12].

Compared with the neonatal mortality rates in China in 1997 (24.4 per 1000 live births), which were provided by UNICEF [13], the rates were significantly lower both in Shenyang's urban areas (3.67 per 1000 live births) and rural areas (6.68 per 1000 live births). Although these rates were considerably lower than those of some Asian countries (e.g., India with 49 per 1000 live births), they were higher than the neonatal mortality rates of Korea (1.8 per 1000 live births) and Japan (2 per 1000 live births) [13]. During the past two decades, according to a UNICEF report published recently, the neonatal mortality rates of urban areas in 2014 (2.39 per 1000 live births) were slightly higher than that of the Republic of Korea (1.7 per 1000 live births).

The major strengthen of the present study was the valuable evidence of time trends of neonatal mortality from one the most important developing countries, China. Given the high reliability of the data published by the Shenyang Women and Children Health Care Centre, the results of this study showed appropriate representativeness. Furthermore, the long observation period provided the possibility to describe the time trends of different causes of neonatal death. Several limitations of this study also should be acknowledged. First, limited by the access of data, we could not present the rate and trends of early neonatal mortality, number of neonatal deaths under age 7 days per 1,000 live births. Moreover, because there was no case of neonatal death of several causes, we could not calculate the PC of these causes (Table 3). Finally, compared to many developed countries, the Shenyang Women and Children Health Care Centre started collecting information in 1992 and were vulnerable to several common technical problems that could jeopardize the quality of the data collected. During the approximate two decades of centre's operation, our centre, as well as the infrastructural development of China, was experiencing a transition. Additionally, though autopsy is the gold standard to confirm the cause of death of neonates, a relatively low rate of autopsy was observed in several developed countries as well as in China. Nevertheless, we have yielded high consistency between autopsy and determination of causes of neonatal deaths which may partly improve the quality of this study.

In conclusion, the neonatal mortality in Shenyang significantly decreased during the time period of 1997 to 2014. The MDG-4 of neonatal death was achieved in urban areas but not rural areas. This issue could be partly attributed to the different rates and time trends of the several categories of neonatal death. The findings of the present study are important for policy makers to gain a better understanding of the fluctuation of neonatal mortality and to take measures to maintain the recent decreasing trends of these causes, thereby reducing the public health burden in China.

## MATERIALS AND METHODS

### Study population

We defined neonatal mortality as death of the neonates from the day of birth through 28 days after birth, and we calculated the total number of deaths among all neonates during the study period [14]. Live birth-neonate death data files were collected by the Shenyang Women and Children Health Care Centre which was a comprehensive care institution and the Centre of Women and Children's Health Care Guidance in the city of Shenyang since 1987. Its catchment area includes five urban districts (He Pin, Shen He, Da Dong, Huang Gu, and Tie Xi) and eight rural districts (Liao Zhong, Kang Pin, Fa Ku, Xin Ming, Shen Bei, Hun Nan, Yu Hong, and Su Jiatun), with a total of 8.1 million inhabitants.

### Data source

The main procedure for gathering data is passive notification of cases, both by public or private hospitals/clinics. The data concerning the cause of neonatal deaths were classified according to the International Classification of Diseases (ICD)-10 codes. We obtained the data regarding neonatal deaths between January 1, 1997 and December 31, 2014. The main categories of neonatal mortality were the following: infectious and parasitic diseases; congenital malformations; diseases of the perinatal period, diseases of the nervous system and sense organs; diseases of the respiratory system; diseases of the digestive system; accident; and other cause of neonatal death, for example endocrine, nutritional and metabolic diseases; blood and hematopoietic organ diseases; circulatory system diseases; urinary system diseases; and leukaemia and other neoplasms [6]. The unknown or missing causes of deaths were also officially classified into other causes of deaths (6). The unknown or missing cause's rates were less than 8% through the seventeen years.

### Statistical analysis

Neonatal mortality rates were calculated for seventeen 1-year time intervals from 1997 to 2014. The annual percentage change (APC) for neonatal mortality rates was used to quantify the time trends [15, 16]. A regression line was fitted to the natural logarithm of the rates, weighted by the number of cases, i.e. y = α + βx + ε, where y = ln (rate) and x = calendar year, and then the APC was calculated as 100 × (e^*β*^−1). The 95% confidence interval (CI) of the APC was calculated by the methods for population-based cancer statistics recommended by the National Cancer Institute [17]. Additionally, we calculated the relative contributions for rate changes to determine the contributions made to the overall trend from categories of death causes [17]. This is done by calculating the percent contribution of each negative regression coefficient to the sum of the negative coefficient, and similarly, percents can be calculated for each positive coefficient [17]. If the overall trend is decreasing, then it is assumed that the percentages for the subgroups with a negative regression coefficient represent their relative contribution to the decreasing trend and are, in effect, a part of the decreasing trend [17]. When grouped by selective causes of neonatal mortality, we only presented the results of the causes that did not have zero death in overall areas during 1997–2014 (septicemia, pneumonia, congenital heart disease, preterm birth and low birth weight, birth asphyxia, intracranial hemorrhage of the newborn, and accidental asphyxia). All analyses were conducted using SPSS for Windows (version 17, SPSS Inc, Chicago, IL, USA). All statistical tests were two-sided, and *P*-values less than 0.05 were considered statistically significant.
